# Relationship between hydrothermal temperatures and structural properties of CeO_2_ and enhanced catalytic activity of propene/toluene/CO oxidation by Au/CeO_2_ catalysts

**DOI:** 10.3389/fchem.2022.959152

**Published:** 2022-09-21

**Authors:** Srisin Eaimsumang, Nuwong Chollacoop, Apanee Luengnaruemitchai, Stuart H. Taylor

**Affiliations:** ^1^ The Petroleum and Petrochemical College, Chulalongkorn University, Bangkok, Thailand; ^2^ Renewable Energy Laboratory, National Energy Technology Center (ENTEC), Pathumthani, Thailand; ^3^ Center of Excellence in Catalysis for Bioenergy and Renewable Chemicals, Chulalongkorn University, Bangkok, Thailand; ^4^ Cardiff Catalysis Institute, School of Chemistry, Cardiff University, Cardiff, United Kingdom

**Keywords:** ceria, gold, VOCs, oxidation, morphology

## Abstract

A simple hydrothermal synthesis of CeO_2_ was implemented to obtain a series of CeO_2_-supported gold (Au) catalysts, used for the total oxidation of propene/toluene/CO gas mixtures and the oxidation of CO. CeO_2_ preparation started from a cerium hydrogen carbonate precursor using a range of different hydrothermal temperatures (HT) from 120 to 180°C. High-resolution transmission electron microscopy, X-ray diffraction, and H_2_-temperature-programmed reduction data indicated that CeO_2_ morphology varied with the HT, and was composed of the more active (200) surface. Following Au deposition onto the CeO_2_ support, this active crystal plane resulted in the most widely dispersed Au nanoparticles on the CeO_2_ support. The catalytic performance of the CeO_2_-supported Au catalysts for both oxidation reactions improved as the reducibility increased to generate lattice oxygen vacancies and the number of adsorbed peroxide species on the CeO_2_ support increased due to addition of Au. The Au catalyst on the CeO_2_ support prepared at 120°C was the most active in both propene/toluene/CO oxidation and independent CO oxidation.

## 1 Introduction

Volatile organic compounds (VOCs) are considered major contributors to air pollution, and they normally consist of a wide range of compounds, including alcohols, aldehydes, ketones, and both aromatic and aliphatic hydrocarbons (Liotta et al., 2008). Anthropogenic emissions of VOCs are often the results of either industrial processes or transportation. In particular, petrochemical processes result in the emission of light hydrocarbons, aromatic compounds, and products of incomplete combustion of organic compounds. Whilst for transportation, dual-fuel engines, running on liquefied petroleum gas (LPG) and diesel, have been developed to enhance engine performance (Ashok et al., 2015); but this engine releases propene, toluene, and carbon monoxide (CO) (Makino et al., 2007). Hence controlling VOC emissions has been extensively investigated as an important issue. Indeed, by 2020, the maximum VOC emission level in European Union countries was stated to be reduced by nearly half compared to the year 2000 (Agency, 2020). Among the physical, chemical, and biological treatments available to reduce VOC emissions, catalytic oxidation is a promising approach, as it can effectively achieve conversion of VOCs into CO_2_, water, and less harmful compounds at lower temperature than thermal oxidation (Kamal et al., 2016).

The high catalytic performance of noble metals (*i.e.*, Pt, Pd, and Rh) in the oxidation of VOCs has been extensively investigated (Patterson et al., 2000). However, despite high catalytic activity at low temperatures, they are expensive and can be deactivated by sintering or poisoning (Song et al., 2020; Guo et al., 2021). Among noble metal catalysts, Pt-based catalysts are known as the most effective catalysts for the oxidation of benzene, while Pd-based catalysts are the most active for the oxidation of toluene, however, in the presence of CO, the temperature required for the catalytic reactions increases by at least 100 °C (Patterson et al., 2000). Gold-based catalysts demonstrate high catalytic activity for CO oxidation under mild conditions of low temperature (Haruta et al., 1993). A CeO_2_-supported Au catalyst (Au/CeO_2_) exhibits a synergistic function for VOC oxidation due to the fact that gold can interact strongly with the CeO_2_ support, resulting in the improvement of gold redox properties, and the catalyst displays superior stability during VOC oxidation (Scirè et al., 2003; Andreeva et al., 2006; Ousmane et al., 2011). In fact, CeO_2_ supports are well known as reducible oxide supports that can participate in oxidation reactions due to their oxygen transfer properties. The redox shift between Ce^4+^ and Ce^3+^ can take place in these supports under oxidizing and reducing conditions (Gennequin et al., 2007), and the redox cycle is accompanied by incorporation of oxygen at vacant oxygen sites. Moreover, the nature of the CeO_2_ support also influences the properties of Au/CeO_2_ catalysts. Indeed, several reports indicate that it is not just the physical properties of CeO_2_ that influence the characteristics of catalysts consisting of metals supported on CeO_2_, including catalytic activity in oxidation reactions, but also the support structural properties, such as morphology and number of cerium centers present on the catalyst’s exposed surface (Hu et al., 2016; Zhang X. et al., 2017; Eaimsumang et al., 2019). Although nanostructured CeO_2_ supports have been synthesized by different approaches, the hydrothermal method has the advantages of simplicity and of affording a high level of control with respect to support size and morphology. Hydrothermal temperature is one of the factors that influences control over CeO_2_ morphology (Carltonbird et al., 2018). It significantly affects thermal energy participating in crystallization phenomena in terms of kinetic (nucleation) and thermodynamic (crystal growth) control, which can determine transformation of morphology as well as structural properties. The morphological change of CeO_2_ influenced by different hydrothermal temperatures was previously studied and evaluated for catalyst’s characteristics relating to the catalytic activity in specific reactions. The correlation between catalytic activity, the amount of cationic Au species in Au/CeO_2_, and the CeO_2_ morphology has been previously recognized (Carltonbird et al., 2018). Au nanoparticles (NPs) were well dispersed on rod-shaped CeO_2_ with dominantly exposed {110} and {100} surfaces. Similarly, a Cu/Ce-Zr-rod preferentially exposed {110} and {100} surfaces, promoting reducibility and providing a higher number of oxygen vacancies than a Cu/Ce-Zr-cube, resulting in a Cu/Ce-Zr-rod exhibiting superior catalytic activity to a Cu/Ce-Zr-cube for toluene oxidation (Dou et al., 2021).

Even though VOC removal via oxidation reactions over Au catalysts has been studied for over a decade (Scirè et al., 2003; Solsona et al., 2006; Shen et al., 2008), the relevant experiments were conducted independently on each reactant, which might not be a practical approach for application to real-life scenarios. The oxidation of propene/toluene/CO mixtures over Au/CeO_2_ catalysts has been previously studied, and the experiments conducted only focused on the effect that Au catalyst preparation had on the catalytic performance (Aboukaïs et al., 2013). The high catalytic performance of Au/CeO_2_ depends on the increased oxygen mobility in the reducible oxide support resulting from Au introduction. Therefore, it is still challenging to fabricate Au NPs and investigate the crucial factors in catalyst preparation that influence the catalytic properties of the nanoparticles for VOC oxidation. The present work studies catalytic performance of Au/CeO_2_ catalysts for the oxidation of VOCs, whereby hexadecyltrimethylammonium bromide (CTAB) was employed as the template for CeO_2_ synthesis (Eaimsumang et al., 2020); in the present study, the temperature of hydrothermal CeO_2_ synthesis was varied to investigate the effect on both the CeO_2_ and Au/CeO_2_ as catalysts for the oxidation of a propene/toluene/CO gas mixture. Moreover, the same catalysts were tested for independent CO oxidation in order to investigate the effect of competing reactions.

## 2 Experimental

### 2.1 Catalyst preparation

The synthesis of the CeO_2_ supports was carried out based on the results of our previous work (Eaimsumang et al., 2020). In detail, an aqueous solution of 6.9 mmol of Ce(NO_3_)_3_·6H_2_O (Sigma-Aldrich, 99%), 0.81 mmol of CTAB (Sigma-Aldrich, 96%), and 27.6 mmol of urea (Sigma-Aldrich, 99%) were added into 30 ml of deionized water under stirring at room temperature; the solution obtained was then stirred at room temperature for 15 min. The resulting solution was transferred into a 100 ml Teflon-lined stainless-steel autoclave reactor and hydrothermally treated in the oven at the desired temperature for 12 h, and subsequently allowed to cool to ambient temperature. The solid product obtained was washed thoroughly with excess deionized water and collected by filtration. The resulting white powder was dried in an oven at 80°C for 24 h and calcined in static air at 500 °C for 10 h. The CeO_2_ support was labeled as CeO_2_X, where X is the hydrothermal temperatures (HT) of synthesis.

The Au/CeO_2_X catalysts (Au loading of 3 wt%) were prepared by a deposition–precipitation (DP) method, implementing the procedure detailed in our previous report (Eaimsumang et al., 2020). Briefly, the dried CeO_2_X was added to an aqueous solution of 2 mmol/L hydrogen tetrachloroaurate (III) acid (HAuCl_4_·3H_2_O; 99.9%, Sigma-Aldrich) under vigorous stirring at room temperature; the mixture obtained was then stirred at room temperature for 1 h. During this time, the pH of the solution was adjusted to 8,9 by adding 0.1 M (NH_4_)_2_CO_3_ solution. The resulting precipitate was washed with excess deionized water until the washings were at pH 7. The precipitate was dried under vacuum at 80°C for 16 h and calcined in static air at 400°C for 4 h.

### 2.2 Catalyst characterization

The crystalline structures of the CeO_2_ supports and Au/CeO_2_ catalysts were analyzed using an X-ray diffractometer system (RINT-2200) with Cu Ka radiation, operated at 40 kV and 30 mA. Diffraction data were obtained over the 2θ range 20°–80°, applying a continuous scan at a rate of 2θ = 5°/min and a scan step of 0.02°. The mean crystallite size (D) of the Au/CeO_2_ catalysts and CeO_2_ supports were calculated from line broadening of the most intense reflections using the Scherrer equation:

D=(0.94λ)/(β⁡cosθ) (1)

where λ is the wavelength of the Cu Kα (0.15406 nm), β is the full width at half maximum of the X-ray diffraction peak, and θ is the Bragg angle of the characteristic peak.

The lattice constant (a) was calculated by the following equation:

$$a=d_{\mathrm{hkl}}/{\left(h^{2}+k^{2}+l^{2}\right)}^{1/2}$$ (2)

where d represents lattice spacing of CeO_2_ and h, k, and l represent the miller indices of the crystallographic plane.

The CeO_2_X morphologies were investigated by field emission gun scanning electron microscopy (FEG-SEM) using a Tescan MAIA3 microscope. The samples were coated with Au:Pt (80:20) with 10 nm thickness. The morphologies and exposed crystal planes of the Au catalysts were identified by high-resolution transmission electron microscopy (HR-TEM) using a JEOL JEM-3100F instrument operated at a 300 kV accelerating voltage. The mean Au particle size distribution (d_Au_) was estimated from TEM images via the following equation:

$$d_{\mathrm{Au}}=\sum n\times d/\sum n$$ (3)

where n is the number of Au particles and d is the measured diameter of Au particles (nm).

The specific surface area of the catalysts was determined by the Brunauer–Emmett–Teller (BET) method from the adsorption of N_2_ at −196°C using a Quadrasorb-evo instrument (Quantachrome). Notably, the catalysts were degassed under vacuum at 200°C for 16 h prior to analysis.

H_2_-temperature-programmed reduction (H_2_-TPR) experiments were carried out using a BELCAT II instrument equipped with a thermal conductivity detector (TCD). A gas mixture consisting of 5.13% H_2_ in Ar (30 ml/min) was used to reduce the sample (0.1 g) contained in a quartz tube reactor. The reduction temperature was raised from 30 to 900°C at a constant heating rate of 10°C/min.

X-ray photoelectron spectroscopy (XPS) analysis was performed using a Kratos Axis Ultra DLD photoelectron spectrometer, equipped with a monochromatic Al Kα radiation operated at 15 kV under a pressure of less than 5 × 10^–7^ Torr. All XPS spectra were corrected by referencing the energies to the C 1 s peak at 284.6 eV. The XPS spectra were analyzed employing CasaXPS software.

Raman spectroscopy was utilized to determine the vibrational modes of the catalysts using a Renishaw InVia Raman spectrometer with a laser excitation line of wavelength 532 nm, at a power of 10 mW. The spectra were recorded at a resolution of 1 cm^−1^.

The actual gold loading for each catalyst was quantified via an approach based on inductively coupled plasma optical emission spectroscopy (ICP-OES) measurements performed on a Perkin Elmer Optima 4300 D, using Ar/N_2_ plasma. The Au/CeO_2_X samples (7.5 mg) were digested in aqua regia (HCl (Aldrich, 37%)/HNO_3_ (Aldrich, 67%): 3/1) at 70 °C under stirring for 20 min. The residual particles in solution were removed by filtration, and the final volume of the solution was subsequently adjusted to 100 ml using deionized water.

### 2.3 Catalytic performance testing

Catalyst performance was measured using a laboratory fixed bed microreactor. Approximately 0.1 g of catalyst was secured in an 8-mm-inner-diameter quartz tube, which was utilized forana the catalytic oxidation of a propene/toluene/CO gas mixture over a reaction temperature ranging between 100 and 450°C under atmospheric pressure. The reactant gas mixture, which consisted of propene 1,000 ppm, CO 1000 ppm, toluene 100 ppm, and 6% O_2_ with N_2_ balance, was fed into the reactor at a constant flow rate of 50 ml/min. The gas hourly space velocity (GHSV) was maintained at 50,000 h^−1^. Reactants and products were investigated by performing qualitative and quantitative analyses using online Fourier-transform infrared (FTIR) spectroscopy employing a calibrated Gasmet process analyzer. The catalyst was allowed to stabilize at each reaction temperature, and repeat analyses made until consistent quantification was obtained, ensuring the catalyst was at steady state operation. The conversion percentage was calculated from the following equation:

$$Conversion\;\left(\%\right)=\frac{C_{i}^{in}-C_{i}^{out}}{C_{i}^{in}}\times 100$$ (4)

where $C_{i}^{in}$ represents the amount of each component in ppm fed into the catalyst, and $C_{i}^{out}$ represents the amount of each component in ppm exiting the catalyst.

The CO oxidation reactions were conducted in a fixed-bed reactor under atmospheric pressure in the presence of a gas mixture consisting of CO 1,000 ppm with 2 vol %O_2_, balanced with He, at a reaction temperature ranging between 25 and 150°C. The GHSV was maintained at 50,000 h^−1^. Once the catalyst attained steady state, the reactants and products (O_2_, CO, and CO_2_) were analyzed by on-line gas chromatography using an Agilent 6890N instrument equipped with a packed carbosphere column and a TCD. The CO conversion was calculated via the following equation:

$$CO\;conversion\;\left(\%\right)=\frac{CO^{in}-CO^{out}}{CO^{in}}\times 100$$ (5)

where CO represents the concentration of CO in ppm.

## 3 Results and discussion

### 3.1 Catalyst characterization

#### 3.1.1 SEM analysis

**FIGURE 1:**
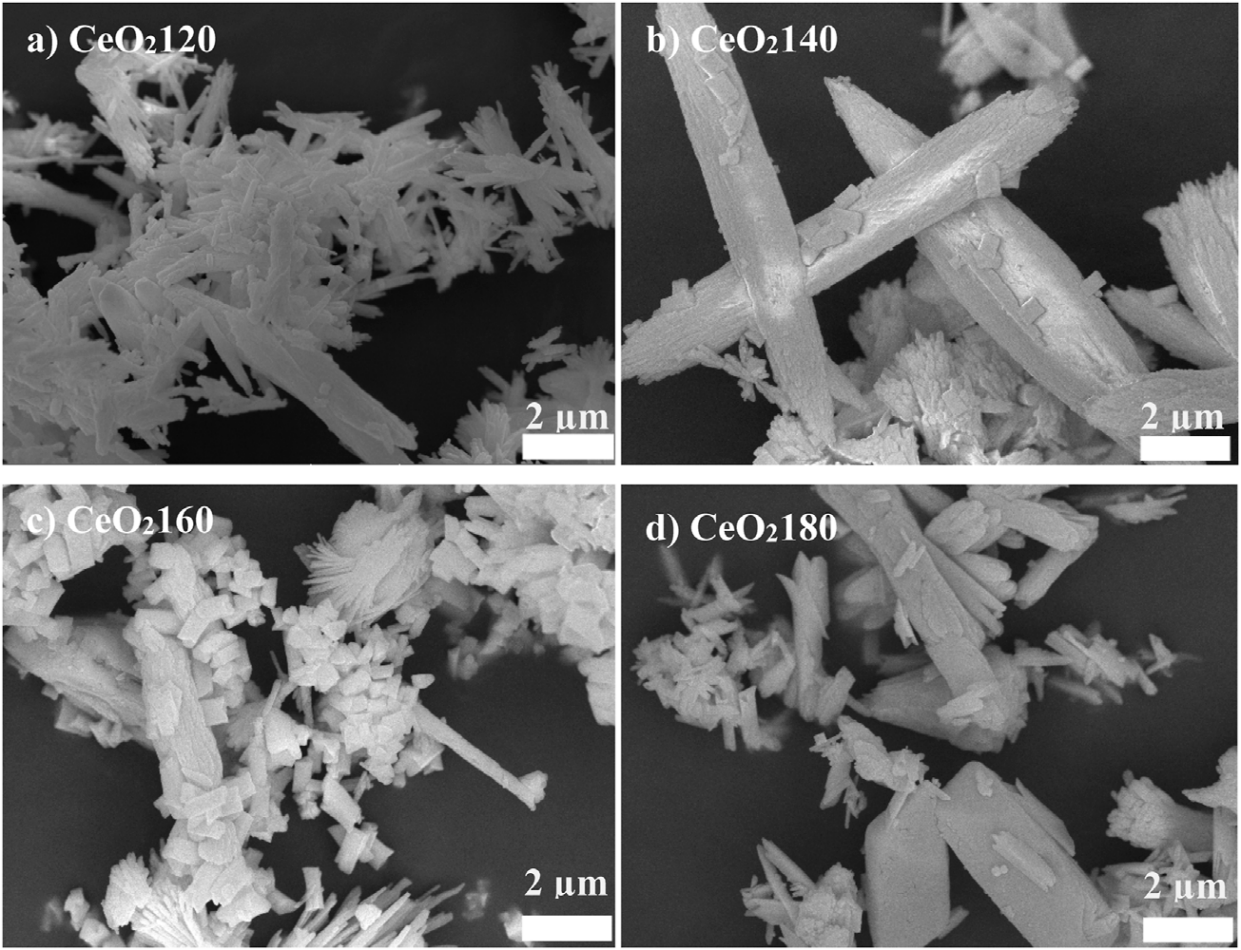
Representative scanning electron microscopy images of **(A)** CeO_2_120, **(B)** CeO_2_140, **(C)** CeO_2_160, and **(D)** CeO_2_180. Scale bar: 2 µm. The reported images are representative of those obtained from at least three such fields of view and two independent samples. CeO_2_X: cerium oxide obtained at a value X for the temperature of the preparatory hydrothermal process.

The morphological features of the CeO_2_ samples were characterized by SEM at a magnification of 300,000. Figure 1 shows SEM images providing information on the morphologies of the CeO_2_ supports prepared after 12 h at different synthesis temperatures. Bundles of rod-like particles were obtained at 120°C. The distribution of the widths (62–113 nm) and lengths (552–1,132 nm) of the rod-like particles are shown in Figure 1A. When the synthesis temperature increased to 140°C, although the bundles of rod-like particles still existed, small cubic plates were also identified on the surface of the rod-like particles (Figure 1B). CeO_2_ prepared at 160 °C consisted of a mixture of rod-like particles and a fraction comprising particles of nonuniform polyhedral morphologies (Figure 1C). By contrast, CeO_2_180 comprised larger particles than CeO_2_120, CeO_2_140, and CeO_2_160, which also exhibited clearer sharp edges (Figure 1D). The particles obtained followed the basic law of thermodynamics of crystallization, meaning that they became larger due to the aggregation of crystals after complete nuclei formation in order to reduce surface energy. The characteristics of the CeO_2_ supports synthesized herein are in agreement with the results reported by (Cui et al., 2010). With respect to the influence of synthesis temperature on the morphology of CeO_2_, rod-like particles were produced at 120 °C HT, while increasing the temperature up to 160 °C resulted in the generation of particles of nonuniform polyhedral shape.

#### 3.1.2 Powder X-ray diffraction analysis

**FIGURE 2:**
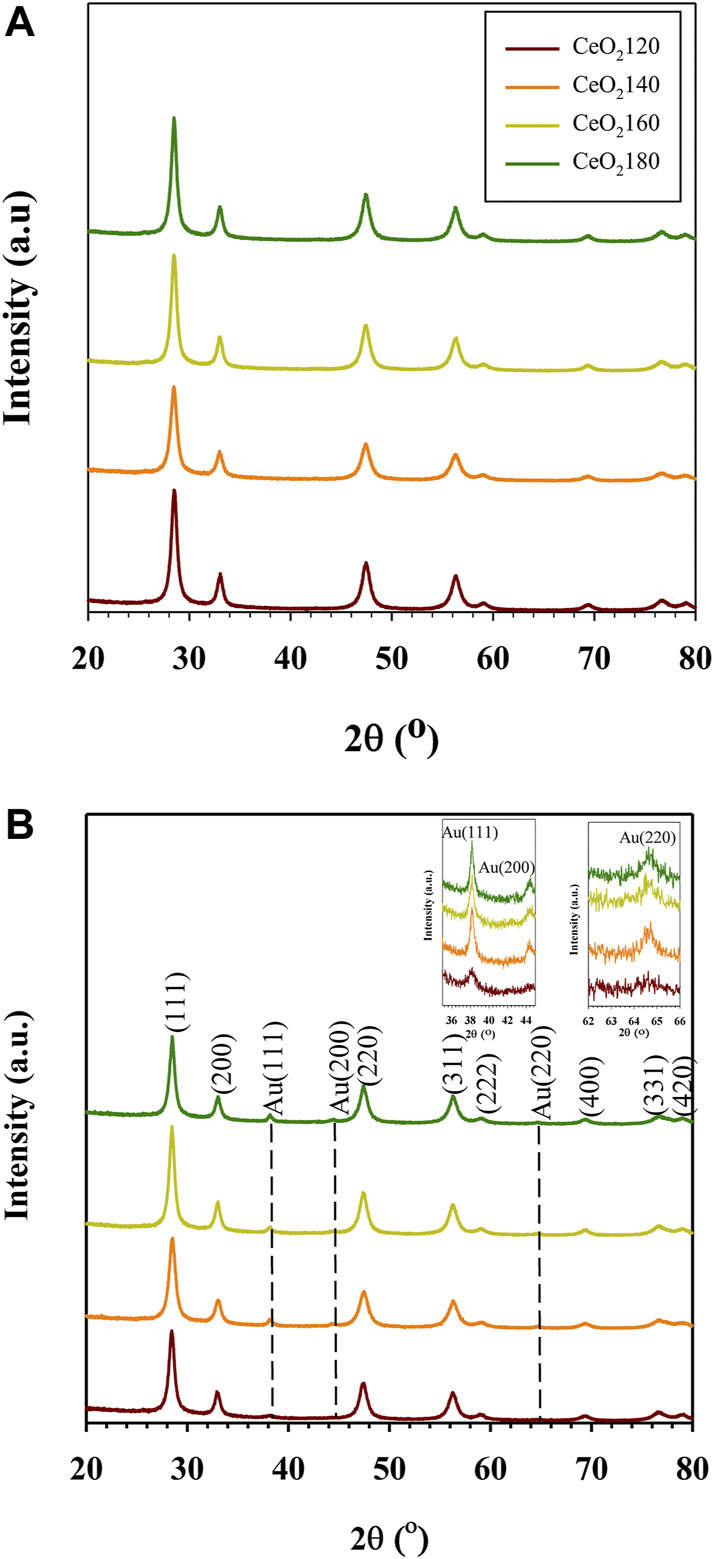
Representative wide-angle X-ray diffraction patterns of **(A)** the synthesized CeO_2_X support and **(B)** the prepared Au/CeO_2_X catalysts. The reported diffractograms are representative of those obtained from two independent samples. CeO_2_X: cerium oxide obtained at a value X for the temperature of the preparatory hydrothermal process; Au/CeO_2_X: CeO_2_X-supported gold catalysts.

Representative X-ray diffraction (XRD) patterns confirmed the fluorite cubic structure of CeO_2_, as indicated by the characteristic peaks due to (111) (200) (220) (311) (222) (400) (311), and (420) lattice planes, referenced to ICDD card number 01–078–5,328 (Figure 2). The average crystallite size was calculated based on the Scherrer equation from the diffraction peak of (111). The crystallite size of CeO_2_ alone increased slightly (from 12.6 to 13.2 nm) as the HT value increased, which was less of an effect on crystallite size than CeO_2_ prepared in the presence of NaOH or ammonia as a precipitant (Carltonbird et al., 2018). Given the fact that the nucleation is *via* initial formation of cerium hydroxycarbonate by reaction with decomposed urea during hydrothermal treatment, whereas the nucleus of cerium IV hydroxide can immediately be precipitated using NaOH or NH_4_OH in the solution at room temperature, the crystal growth rate is substantially faster for the hydroxide precipitation-based method than for the urea-based one.

Notably, all members of the Au/CeO_2_X catalyst series exhibited the presence of an Au crystalline phase. The Au (111) diffraction peak was detected at a 2θ value of 38.2°. In contrast, the crystal planes of Au (200) and Au (220) were detected at 2θ values of 44.3° and 64.7°, respectively. As evidenced from the data listed in Table 1, Au/CeO_2_120 was characterized by the smallest Au crystallite size [D_Au(111)_], with a value of 8.6 nm, which is between 1.8 and 2.8 times smaller than the corresponding value for the other Au/CeO_2_X catalysts. The other catalysts were characterized by very large Au (200) and Au (220) crystallite sizes. Unexpectedly, the Au (200) and Au (220) lattice planes were undetectable for Au/CeO_2_120. Moreover, the addition of Au to the CeO_2_ resulted in an increase in the lattice constant, pointing towards an increase of defects in the CeO_2_ structure, as the ionic radius of Ce^3+^ is larger than that of Ce^4+^ (Vinothkumar et al., 2019).

**TABLE 1:** Physicochemical properties of the synthesized CeO_2_X supports and Au/CeO_2_X catalysts.

Sample	S_BET_ (m^2^/g)	Pore Volume (cc/g)	^a^D_Ce(111)_ (nm)	Lattice Constant (nm)	^b^Au Loading (wt%)	^a^D_Au(111)_ (nm)	^a^D_Au(200)_ (nm)	^a^D_Au(220)_ (nm)	^c^D_Au_ (nm)	^d^Defect (A_600_) (%)	^d^Defect (A_1180_) (%)
CeO_2_120	98	0.12	12.9	0.5411	N/A	N/A	N/A	N/A	N/A	1.12	2.20
CeO_2_140	92	0.11	12.6	0.5416	N/A	N/A	N/A	N/A	N/A	1.05	2.65
CeO_2_160	106	0.10	13.0	0.5414	N/A	N/A	N/A	N/A	N/A	1.12	2.94
CeO_2_180	105	0.13	13.2	0.5418	N/A	N/A	N/A	N/A	N/A	1.18	3.40
Au/CeO_2_120	60	0.10	13.4	0.5431	2.1	8.6	-	-	10.8	3.68	-
Au/CeO_2_140	78	0.09	12.5	0.5422	2.4	16.4	2.7	16.6	22.7	2.64	-
Au/CeO_2_160	82	0.10	13.2	0.5425	2.2	15.6	4.4	16.4	22.8	3.81	-
Au/CeO_2_180	62	0.13	13.7	0.5419	2.5	23.9	20.2	19.9	35.2	4.34	-

a Crystallite size of CeO_2_ or Au calculated based on the relevant characteristic peaks of the X-ray diffraction patterns.

b Actual Au loading determined by inductively coupled mass spectrometry.

c Mean Au particle size estimated based on approximately 75 particles from transmission electron microscopy images.

d Defect concentration calculated by the proportional area of the vibrational bands at 600 and 1,180 cm^−1^ Observed in the relevant Raman spectra.

#### 3.1.3 Determination of surface area and pore size

**FIGURE 3:**
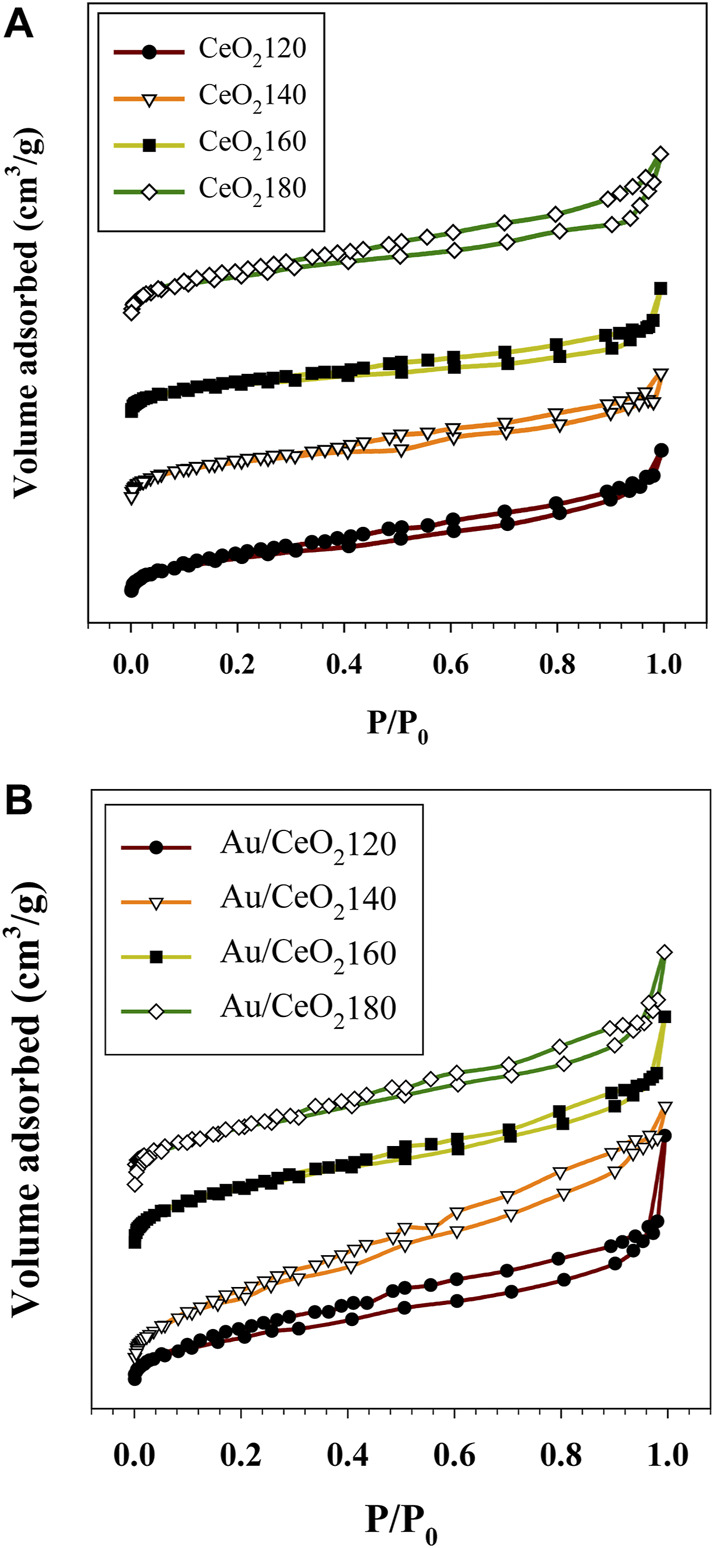
Representative N_2_ adsorption–desorption isotherms of **(A)** the synthesized CeO_2_X supports and **(B)** the prepared Au/CeO_2_X catalysts. The reported isotherms are representative of those obtained from two independent samples. CeO_2_X: cerium oxide obtained at a value X for the temperature of the preparatory hydrothermal process; Au/CeO_2_X: CeO_2_X-supported gold catalysts.

The N_2_ adsorption and desorption isotherms for the supports and the relevant catalysts are reported in Figure 3. According to the IUPAC system, they exhibit the features of type II isotherms, and they are characterized by a steep slope due to micropore filling occurring at relatively low N_2_ pressure. In addition, the capillary condensation, representing a hysteresis loop, occurred over a wide range of relative pressure values. This observation is indicative of a complex pore structure of the material. The hysteresis loop appeared to be largest in the case of CeO_2_180, which implied a nonuniform pore structure and broader pore size distribution for this support than for the others. The parent ceria samples exhibited specific surface areas between 92 and 106 m^2^/g. The BET surface areas of the CeO_2_X supports increased with the HT value, corresponding to more rapid hydrolyzation of urea (Cui et al., 2010), and also accelerated hydration of the cerium cation with increasing HT (Wang and Lu, 2002). This process resulted in a large number of CeO_2_ nuclei produced by a large amount of carbonate ions reacting with hydrated cerium cations.

In the case of the Au catalysts, the surface area substantially decreased in comparison with pure CeO_2_, with values ranging between 60 and 82 m^2^/g. On the other hand, the pore volume of ceria (0.11–0.13 cc/g) was slightly different in the case of supports obtained at different HT values. Moreover, the nonuniform pore size distribution of CeO_2_ had values between 1.9 and 11.1 nm, indicating mixed micro and mesoporosity (Supplementary Figure S1). The pore size distribution after Au incorporation was altered from that of the pure ceria. The initial derivative adsorption started at a pore size of approximately 15 nm for all catalysts, except for Au/CeO_2_140, that continued to show pores remaining in the micropore range.

#### 3.1.4 Catalyst morphologies from HR-TEM analysis

**FIGURE 4:**
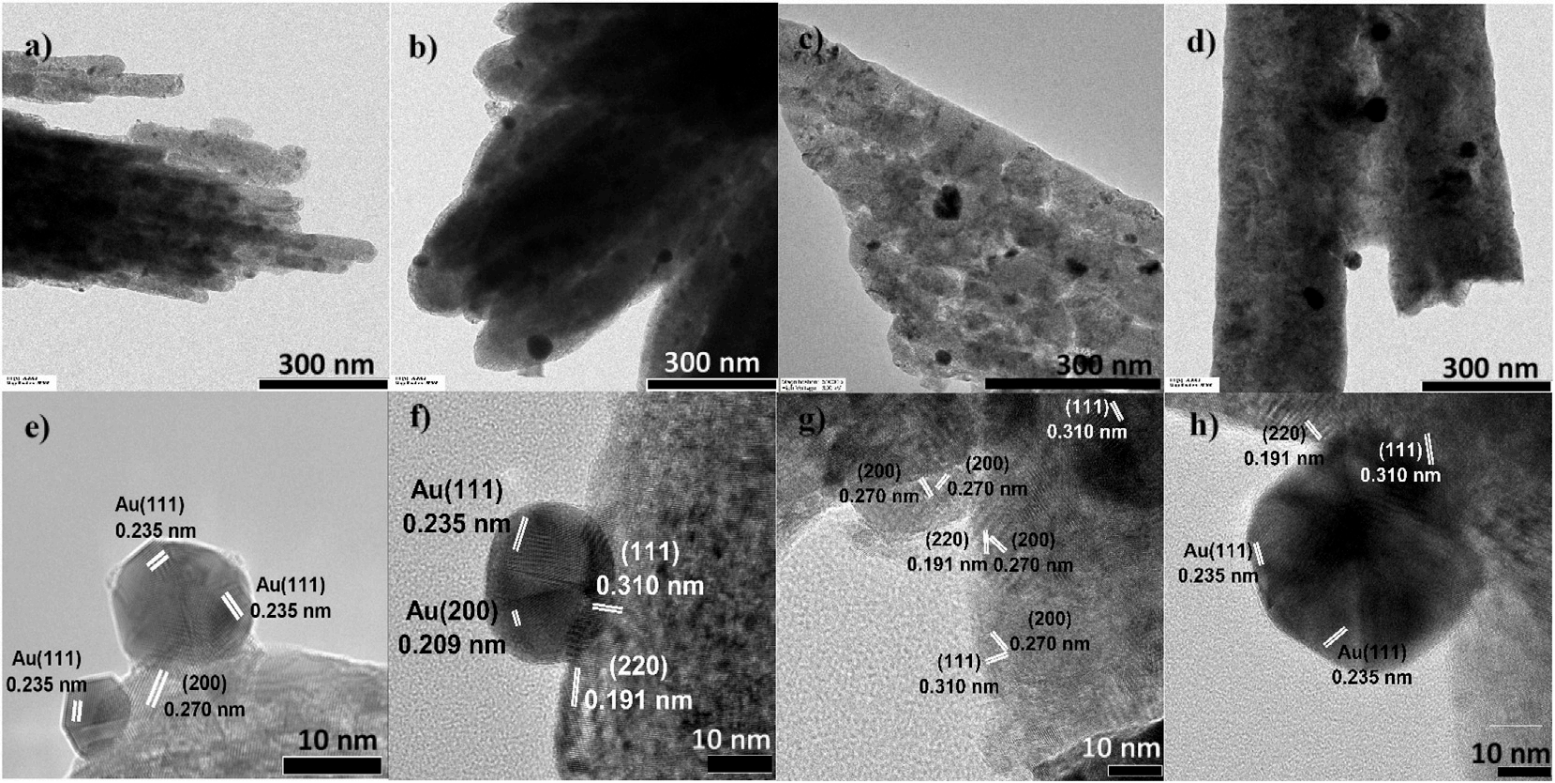
Representative high-resolution transmission electron microscopy images of **(A,E)** Au/CeO_2_120 **(B,F)** Au/CeO_2_140 **(C,G)** Au/CeO_2_160, and **(D,H)** Au/CeO_2_180. CeO_2_X: cerium oxide obtained at a value X for the temperature of the preparatory hydrothermal process; Au/CeO_2_X: CeO_2_X-supported gold catalysts.

The different CeO_2_ morphologies were established based on representative HR-TEM images, and the collected HR-TEM data were observed to be in agreement with the SEM data (see Figure 1). The particle sizes increased at higher HT values, suggesting that crystalline particles underwent aggregation from a lot of nuclei precipitated by excess urea precipitant concentration (urea:Ce^3+^ = 4:1). The Au particle size distribution was measured from the HR-TEM images. The average Au particle size differed substantially between catalysts, as can be evidenced from the data in Table 1. The Au particle sizes were observed to increase with the HT value of the CeO_2_ support. Au/CeO_2_120 comprised Au particles of sizes that were 3.3-fold smaller than those present in Au/CeO_2_180, an observation attributed to the greater degree of Au dispersion on the more unstable CeO_2_ (200) surface. Figures 4E–H shows the lattice fringes of all catalysts, which can be used to calculate the d-spacings. The most unstable (220) and (200) surfaces of CeO_2_, corresponding to d-spacings of 0.191 and 0.270 nm, respectively, were observed at the edge of the particles. Au/CeO_2_160 was characterized by multiple exposed surfaces, such as CeO_2_(220) and CeO_2_(200), near the edges of the particles, and the nearby surface was a CeO_2_(111) surface. Figure 4E shows an image of the crystal Au (111) surface located on the CeO_2_(200) surface at the tip of the exposed rod. Whereas, Au (111) was deposited on the edge of particles exposing the CeO_2_(111) surface with the d-spacing of 0.310 nm (Figure 4F), and CeO_2_(220) with d-spacing 0.191 nm existed near to the Au crystal in the case of Au/CeO_2_140 and AuCeO_2_180.

#### 3.1.5 H_2_-TPR studies

**FIGURE 5:**
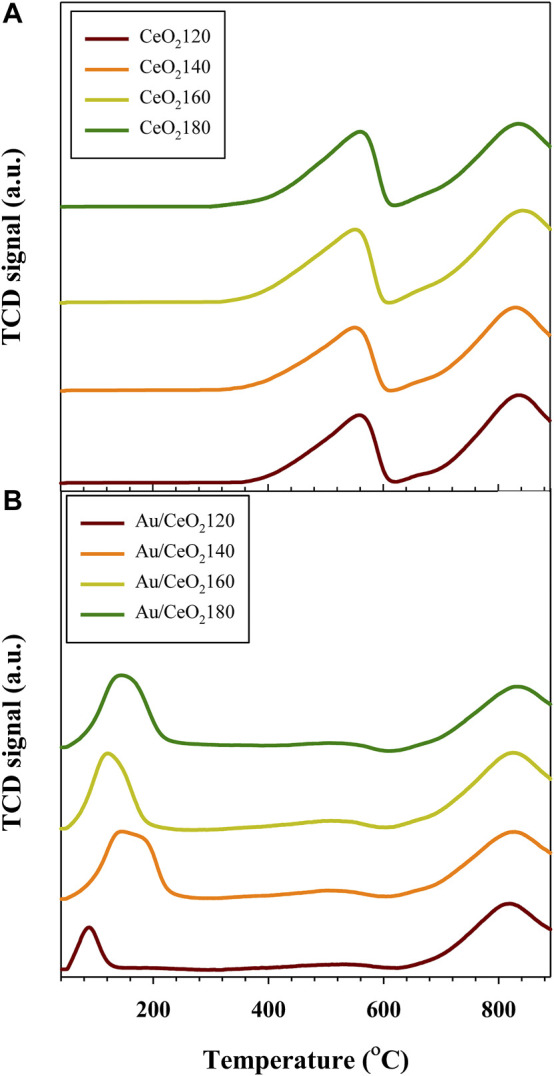
Representative H_2_-temperature-programmed reduction profiles of **(A)** CeO_2_X supports and **(B)** Au/CeO_2_X catalysts. CeO_2_X: cerium oxide obtained at a value X for the temperature of the preparatory hydrothermal process; Au/CeO_2_X: CeO_2_X-supported gold catalysts; TCD: thermal conductivity detector.

The redox behavior of the Au/CeO_2_X catalysts and of the CeO_2_X supports were measured using H_2_-TPR experiments (see Figure 5). According to the H_2_-TPR profiles of the different CeO_2_ supports, the H_2_ reduction of the CeO_2_ surface takes place at 551–561 °C, and subsequently precedes the bulk reduction at higher temperatures of 830–842°C (Carltonbird et al., 2018). Estimating reducibility based on H_2_ consumption data, the CeO_2_X samples had slight differences in the extent of surface reducibility, as evidenced from the data listed in Table 2. After Au deposition, the reduction temperatures of both the surface and bulk shifted to lower values, as evidenced from Figure 5B, indicating the existence of an interaction between the metal and support. Three major reduction peaks were identified. The surface reduction temperature was substantially reduced to between 89 and 147°C, indicating significantly easier oxygen migration in the catalysts than in the bare supports. This is due to the weakening of the Ce–O bonds brought about by the strongly binding gold species (Deng et al., 2007; Sakwarathorn et al., 2011). Au/CeO_2_120 exhibited the lowest reduction temperature (89°C), corresponding to the reduction of nanosized gold oxide and oxygen species located at the interface between Au and CeO_2_ (Ousmane et al., 2011). However, this catalyst also displayed the lowest H_2_ uptake, which might be related to this catalyst having the smallest BET surface area. The small Au NPs could strongly bind onto the CeO_2_ surface, resulting in a stronger interaction between Au and the support. Consequently, the greater ease of reduction temperature due to more readily reduced labile oxygen from high Au dispersion. A similar reduction temperature (94°C) has been reported for Au/CeO_2_ nanorods (Si and Flytzani-Stephanopoulos, 2008). The other Au/CeO_2_ catalysts exhibited values for the lowest reduction temperature in the 121–147°C range; moreover, the extent of H_2_ uptake on the catalyst surfaces can be ordered as follows for the various Au catalysts (Table 2):

$$\mathrm{Au}/\mathrm{Ce}O_{2}\mathrm{140}=\mathrm{Au}/\mathrm{Ce}O_{2}\mathrm{180}>\mathrm{Au}/\mathrm{Ce}O_{2}\mathrm{160}>\mathrm{Au}/\mathrm{Ce}O_{2}\mathrm{120}$$ (6)

**TABLE 2:** Reducibility of the CeO_2_X and Au/CeO_2_ catalysts as inferred from the results of H_2_-temperature-programmed reduction experiments.

Sample	Reduction Temperature (°C)			H_2_ Uptake (mmol/g)		
	T_1_	T_2_	T_3_	T_1_	T_2_	T_3_
CeO_2_120	-	559	839	0	0.50	1.09
CeO_2_140	-	551	830	0	0.53	1.12
CeO_2_160	-	551	842	0	0.56	1.27
CeO_2_180	-	561	836	0	0.57	1.03
Au/CeO_2_120	89	527	819	0.16	0.06	0.99
Au/CeO_2_140	146	503	828	0.46	0.11	1.11
Au/CeO_2_160	121	507	825	0.35	0.08	1.06
Au/CeO_2_180	145	506	831	0.46	0.04	0.84

The surface-capping oxygen of CeO_2_ was more easily reduced at lower temperatures (503–527°C) as compared to pure CeO_2_ (551–561°C). Interestingly, the reduction peak of bulk oxygen also shifted to a lower temperature, indicating that the deposited Au centers can bind bulk oxygen effectively, thereby weakening the Ce–O bond in the bulk of the material. The highest shift in reduction temperature of bulk oxygen was ascribed to Au/CeO_2_120 and Au/CeO_2_160, with decreases in temperature of 20 and 17°C, respectively. This increasing oxygen mobility might result from charge transfer from an Au adatom to the stoichiometric CeO_2_(111) surface, as observed by the presence of Au^+1^ species on the catalyst surface by XPS. The addition of Au should cause an increase in reducibility, due to the formation of Au–O–Ce bonds. This would generate anion vacancies adjacent to Au nanoparticles and then enhanced redox properties. However, our Au catalysts characterized by XRD presented very large Au particle sizes, the said particles might cover the micropore structure of CeO_2_. Moreover, the vacant sites of CeO_2_ were likely to be filled by the deposited Au NPs leading to a decrease in the amount of exposed surface oxygen.

#### 3.1.6 XPS analysis

The surface chemical composition and the chemical states of the elements present on the catalyst surface were investigated by XPS. Based on the data listed in Table 3, the Au content of the catalysts ranged from 0.50 to 0.66%, and decreased in the following order:

$$\mathrm{Au}/\mathrm{Ce}O_{2}\mathrm{180}>\mathrm{Au}/\mathrm{Ce}O_{2}\mathrm{140}>\mathrm{Au}/\mathrm{Ce}O_{2}\mathrm{120}>\mathrm{Au}/\mathrm{Ce}O_{2}\mathrm{160}$$ (7)

**TABLE 3:** Surface elemental composition and chemical states of the Au/CeO_2_X catalysts as inferred by X-ray photoelectron spectroscopy analysis.

Sampe	Au (at%) (wt%)	Ce (at%)	O (at%)	O_latt_ (%)	O_defect_ (%)	O_OH_ _ad_ (%)	Ce^3+^ (%)	Au^0^ (%)	Au^+^ (%)	Au^3+^ (%)	Au^n+^ (%)
Au/CeO_2_120	0.56 (1.88)	32.63	66.81	58.1	35.9	6.0	21.8	50.3	25.2	24.6	49.7
Au/CeO_2_140	0.65 (2.18)	29.96	69.39	66.9	29.1	4.8	17.1	79.7	11.8	8.6	20.3
Au/CeO_2_160	0.50 (1.68)	31.07	68.43	61.4	34.1	4.5	15.7	75.1	14.0	11.0	24.9
Au/CeO_2_180	0.66 (2.22)	32.86	66.48	68.2	26.7	2.1	15.7	79.9	11.9	8.2	20.1

Most Au was dispersed on the surface, rather than in the bulk of the support; in fact, 76.9–91.5% of the actual Au loading was identified on the surface of the support, based on measured total Au content by ICP-OES. Based on the large size of the Au crystalline particles, one can infer that the said particles tend to concentrate on the surface of the support, because most ceria pores are less than 2.5 nm in size (Supplementary Figure S1). Au/CeO_2_120 had relatively low Au content on the catalyst surface, suggesting greater Au NP dispersion and small Au crystal size. Whereas Au/CeO_2_180 had the highest Au content on the surface because dominant Au-Au coordination is an indicator of high Au crystallinity and Au particle size. However, Au/CeO_2_160 had the lowest Au content on the surface, suggesting lower Au dispersion on the surface and Au NPs partially deposited in the bulk of the catalysts. This correlated with the TEM images (Figures 4C,G), which d-spacing 0.235 nm of crystal Au(111) did not appeared for the Au/CeO_2_160 catalyst. The deposition of Au might locate under the subsurface of CeO_2_.

**FIGURE 6:**
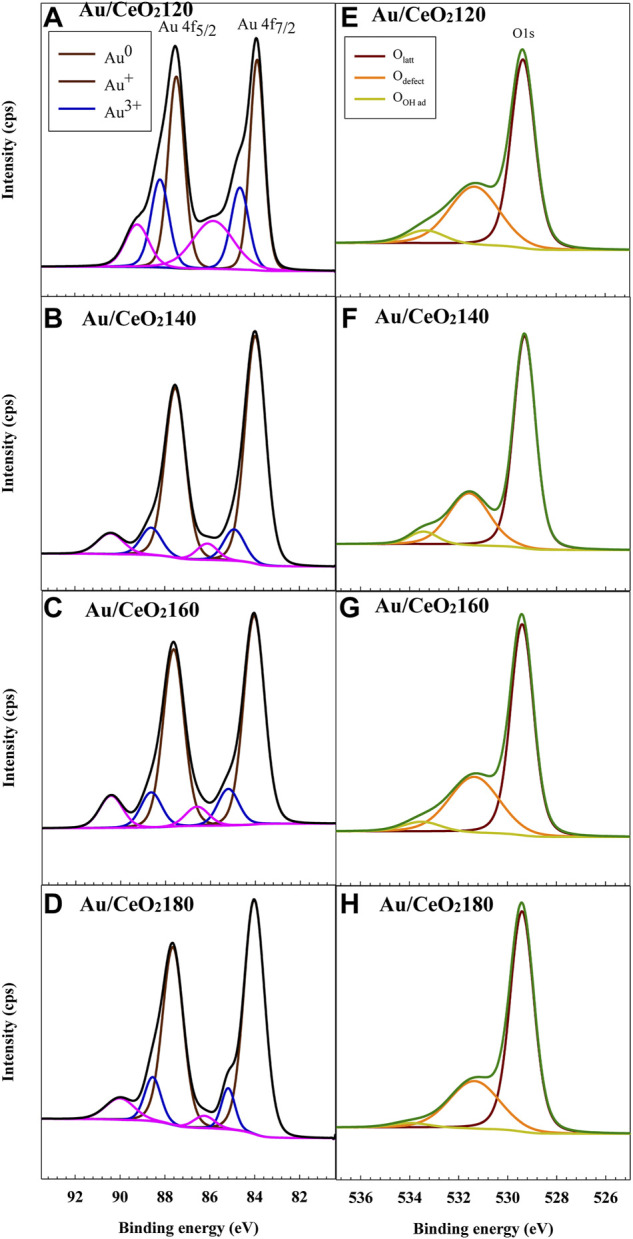
Representative **(A–D)** Au 4f and **(E–H)** O 1s X-ray photoelectron spectra of the Au/CeO_2_X catalysts. CeO_2_X: cerium oxide at a value X for the temperature of the preparatory hydrothermal process; Au/CeO_2_X: CeO_2_X-supported gold catalysts.

The three oxidation states of Au were categorized based on the characteristic peaks of spin–orbit splitting of Au4f _5/2_ and Au4f _7/2_, as illustrated in Figures 6A–D. The well-defined peaks at binding energy (BE) values of 83.8 and 87.5 eV were attributed to the metallic Au (Au^0^) for Au4f_5/2_ and Au4f_7/2_ core levels, respectively. By contrast, the peaks at BE values of 84.9 and 88.7 eV were ascribed to the cationic gold state (Au^+^) (Eaimsumang et al., 2020). The typical peak of Au^3+^ was observed at BE values of 86.3 and 89.9 eV (Pireaux et al., 1984). A surface chemical shift of ionic Au species takes place, which generally reflects the initial state change in electrostatic potential in the atomic core region (Heimann et al., 1981). These Au shifts are partly a consequence of s–d re-hybridization effects at the surface of the catalysts, leading to a shift of surface d-band and 4f levels toward the Fermi level (Desjonqueres et al., 1980; Heimann et al., 1981). For Au/CeO_2_120, the characteristic peaks of Au^+^ and Au^3+^ were slightly shifted to lower BE values by about 0.4 and 0.7 eV, respectively. These shifts are caused by attracting electrons from oxygen vacancies to Au NPs in the presence of X-ray exposure (Kruse and Chenakin, 2011). On the other hand, a slight shift to higher BE values was observed for the Au^n+^ peak in the case of Au/CeO_2_140 and Au/CeO_2_160, which indicated the electron density was located under the subsurface.

Based on a quantitative analysis of the Au species (see Table 3), Au^0^ was the dominant species in all Au/CeO_2_ catalysts, except for Au/CeO_2_120, wherein Au^0^ accounted only for 50.3% of the Au. By contrast, the fraction of the cationic Au species varied among the catalysts. Au/CeO_2_120 exhibited the highest proportion of Au^+^ and Au^3+^ (25.2 and 24.6%, respectively). The Au^3+^ fraction varied between 8.2 and 11.0% in the other three catalysts. Au species composition was observed to be related to Au particle size, and also resulted from an atomic orientation of the Au structure. The smaller the Au particle, the stronger the interaction between Au and the oxygen atom of CeO_2_, and the more prevalent the ionic Au species. Au^0^ existed on the topmost layer of Au nanoparticles, whereas cationic Au was located as layers underneath the nanoparticle, between Au^0^ and the interface between Au and CeO_2_ as reported in a model of the gold–ceria interface (Longo et al., 2012; Guan et al., 2015).

The oxygen species found on the surface of the catalysts were identified by O1s XPS (Figures 6E–H). Three major peaks were observed at BE values of 529.0, 531.0, and 533.0 eV, which were regarded as the characteristic peaks of lattice oxygen and oxygen vacancies and/or the hydroxyl group, respectively. The composition of surface oxygen species was also calculated based on the relative O 1s peak areas. Defects were defined as non-stoichiometric ceria (*i.e.*, CeO_2−x_), whose presence depended on the population of oxygen vacancies. The defect composition differed significantly between samples. The concentration of defects was higher for Au/CeO_2_120 and Au/CeO_2_160, 35.9 and 34.1%, respectively. On the other hand, Au/CeO_2_140 and Au/CeO_2_180 exhibited lower defect concentrations. The presence of cationic Au species contributed to the appearance of defects, because the deposition of Au onto the ceria support resulted in the emergence of oxygen vacancies around the Au atom (Scirè et al., 2003).

The oxidation state of Ce was determined based on a 10-peak fitting of the Ce3d spectra (Supplementary Figure S2). Six peaks, appearing at approximately 882.0, 889.0, 898.3, 900.5, 907.7, and 916.5 eV, were assigned to Ce^4+^. Moreover, the two spin–orbit-coupled doublets of Ce^3+^ appeared at BE values of 880.5, 885.7, 899.7, and 902.3 eV. The presence of Ce^3+^, as opposed to Ce^4+^ only for stoichiometric CeO_2_, in ceria enabled us to infer the existence of oxygen vacancies on the surface of the CeO_2_ support. Quantitative estimation of the Ce^3+^ and Ce^4+^ on the surface was calculated from the total integrated area of the 10 Gaussian fitting peaks (6 and 4 peaks for Ce^4+^ and Ce^3+^ species, respectively), using the ratio of these peak areas (Ce^3+^/Ce^3+^+Ce^4+^) Au/CeO_2_120 was determined to be the catalyst with the highest concentration of Ce^3+^ (21.8%), and it also exhibited the highest concentration of surface oxygen vacancies. This is in agreement with the active role of CeO_2_, as oxygen mobility and electron transfer between Au and CeO_2_ are responsible for the catalytic activity (Andreeva et al., 2006).

#### 3.1.7 Raman spectroscopy investigations

**FIGURE 7:**
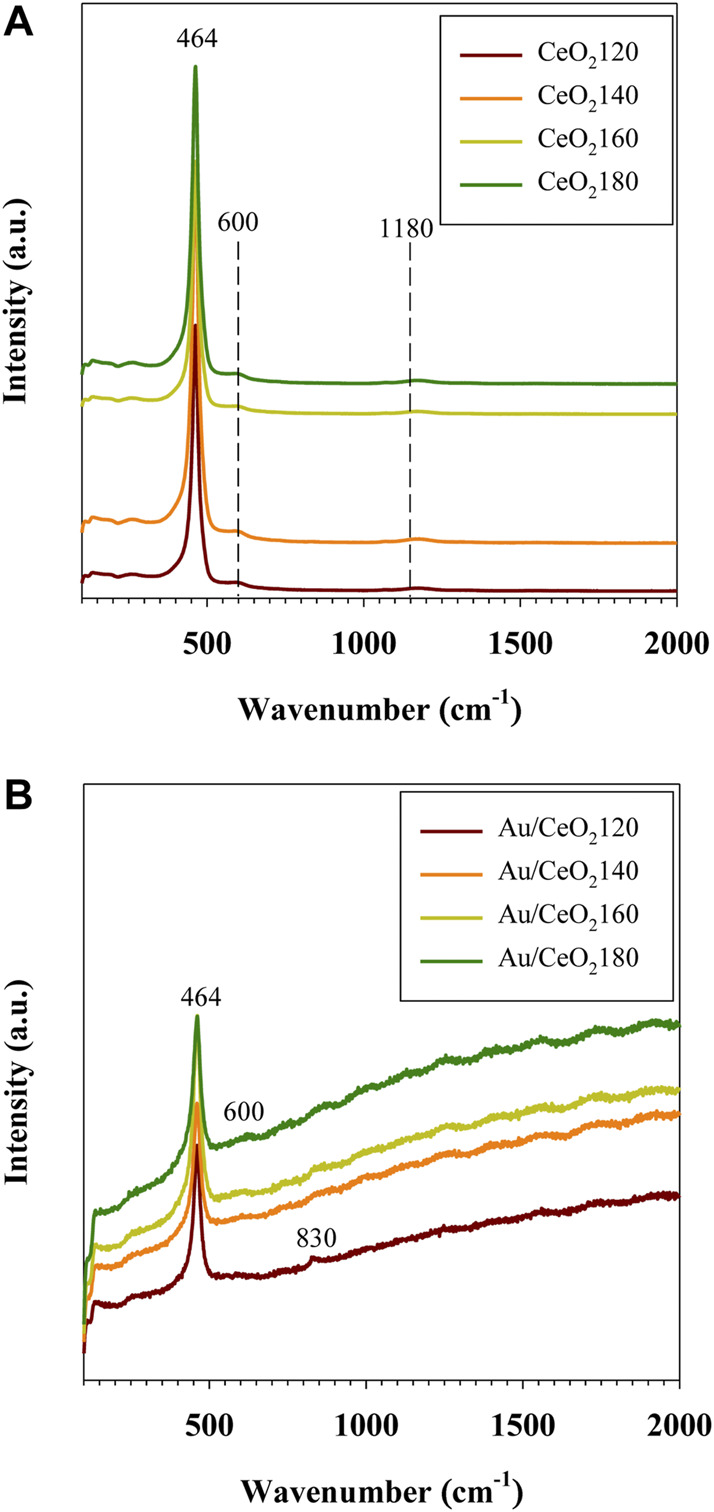
Representative Raman spectra of **(A)** CeO_2_X supports and **(B)** Au/CeO_2_X catalysts. CeO_2_X: cerium oxide obtained at a value X for the temperature of the preparatory hydrothermal process; Au/CeO_2_X: CeO_2_X-supported gold catalysts.

Raman spectroscopy was employed to investigate the vibrational properties of the CeO_2_X supports and the corresponding Au/CeO_2_X catalysts, representative Raman spectra are reported in Figure 7. The band at 250 cm^−1^ wavenumbers was observed, and it was assigned to a vibrational surface of the clean CeO_2_(111) (Filtschew et al., 2016). The intense F_2g_ band at 464 cm^−1^, observed for both CeO_2_X supports and Au/CeO_2_X catalysts, was attributed to the symmetric vibrations of oxygen surrounding the cerium ions in the fluorite-type crystal lattice. The band at 600 cm^−1^, observed for both CeO_2_X supports and Au/CeO_2_X catalysts, is attributed to the presence of oxygen defects in association with cerium ions in the reduced (Ce^3+^) state (Schilling et al., 2017). The weak band at 1,180 cm^−1^, present for CeO_2_X supports and Au/CeO_2_X catalysts, is attributed to the second-order longitudinal optical mode of oxygen defects (Zhang R. B. et al., 2017). However, the defect concentration (A_600_ + A_1180_) in the Au/CeO_2_X catalysts increased with the value of the HT parameter of the parent ceria, as illustrated by the data listed in Table 1. The increase in defect concentration was in line with the enlarged lattice constant as determined by XRD experiments, due to the presence of oxygen vacancies or anion-Frenkel defects (Filtschew et al., 2016). The deposition of Au on CeO_2_ causes a distortion in the oxygen lattice, resulting in asymmetric Raman profiles due to changes in the vibrational properties (Figure 7). Indeed, although the F_2g_ bands were still observed at 464 cm^−1^ in the case of the Au/CeO_2_X catalysts, they were slightly broader than their counterparts observed for the CeO_2_X bare supports, in accordance with the increase in the ease of the lattice oxygen vibration associated with the incorporation of Au, as a result of the weakening of the Ce–O bonds. The defect band at 600 cm^−1^ was broader in the spectra of the Au/CeO_2_X catalysts than in the spectra of the CeO_2_X bare supports, as a result of the appearance of an asymmetric vibrational band after the addition of Au. Interestingly, the band at 832 cm^−1^, which is due to the O=O bond stretching vibration of peroxide groups (O_2_^2−^) present on reduced ceria, was only detected in the Raman spectrum of Au/CeO_2_120. This band originates from the adsorption of molecular oxygen on two-electron defects of ceria (Pushkarev et al., 2004). Its presence indicated that the introduction of Au can promote the formation of peroxide species bound to Ce^4+^. The presence of Au species results in easier formation of oxygen defects on the CeO_2_ surface at the boundary of Au particles, which was assigned to the reduction of ionic gold species and surface ceria in close contact with metallic Au. Notably, this particular phenomenon was also observed in the case of Au/TiO_2_ catalysts (Ousmane et al., 2011). In terms of defect concentration in CeO_2_, the presence of the peroxide species was associated with only a slight enhancement for Au/CeO_2_120 from 3.32 to 3.68% (see Table 1), which likely correlated with the Au particle size effect. The addition of Au should have increased the concentration of oxygen vacancies due to the formation of the Au–O–Ce bonds. If this were the case, anion vacancies should be preferentially found in locations adjacent to Au nanoparticles. However, other Au catalysts consisted of very large Au crystal sizes (>10 nm). Another possible reason is that the vacancy sites of CeO_2_ were the nucleation site for Au deposition.

### 3.2 Effect of HT on CeO_2_ and AuCeO_2_ structural properties and catalysis

**FIGURE 8:**
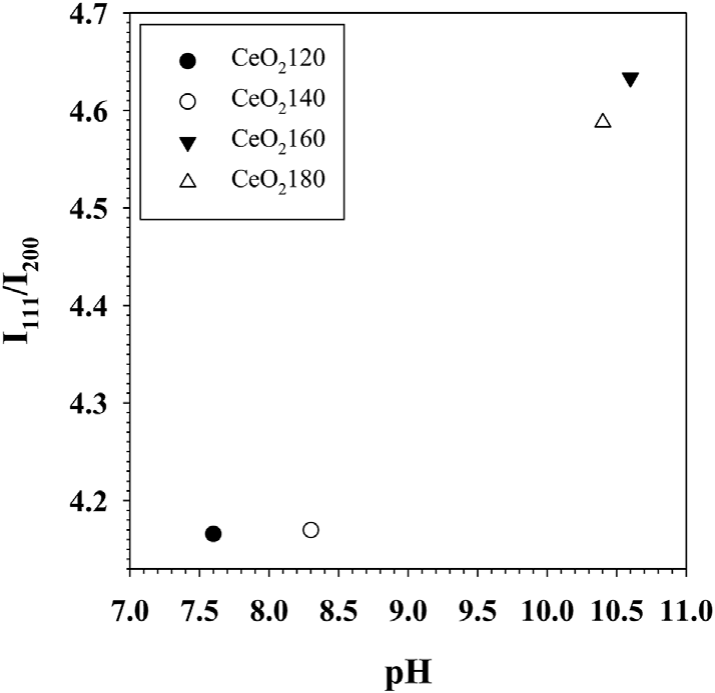
Scatter plot of the crystal phase ratio of (111)/(200) in CeO_2_X supports *versus* the pH of solution measured after hydrothermal aging. CeO_2_X: cerium oxide obtained at a value X for the temperature of the preparatory hydrothermal process.

The hydrothermal temperature of CeO_2_ synthesis has an impact on the physicochemical properties of the ceria. Although crystallite size generally increased slightly as the HT increased between 120 and 180°C, the crystal size of CeO_2_140 was slightly lower than that of CeO_2_120. This phenomenon can take place in the case of CeO_2_ derived from cerium hydroxycarbonate. In this case, larger rod-shaped particles might inhibit the release of CO_2_ from the bulk of the material to the exterior during calcination. Hence, the transformation of Ce(OH)CO_3_ into CeO_2_ is not kinetically favored in large Ce(OH)CO_3_ particles. Therefore, a decrease in the degree of crystallinity of the obtained CeO_2_ is observed (Wang and Lu, 2002). As suggested by XRD data, the value of the I_111_/I_200_ intensity ratio generally increased with the HT, as evidenced from the data in Figure 8. This indicates that in a polyhedral morphology several facets of the material were exposed, which consisted of the more stable crystal plane of (111) than planes exposed for other morphologies. By contrast, a rod-like morphology has preferential longitudinal growth, exhibiting more of the (200) crystal plane. However, the ratio of I_111_/I_200_ increased significantly as the HT value increased from 140 to 160 °C, which predominantly favors the CeO_2_(111) plane over CeO_2_(200) (Ghosh et al., 2010). The substantial difference in the value of the I_111_/I_200_ ratio between CeO_2_160 and CeO_2_140 was ascribed to the change from the rod-like to the polyhedral morphology. At elevated temperature, the morphological change was the result of the particle growth rate becoming higher than the nucleation rate (Chen and Chang, 2005). Moreover, the partial pressure inside the hydrothermal vessel increased at higher temperature causing the particles to aggregate with many tiny grains and more unstable surfaces fused together according to thermodynamics, which results in more exposure of the (111) surface. Hence, the morphological change was accompanied by an increase in particle size between CeO_2_160 and CeO_2_140, which was evident in the relevant TEM images. At high HT values, the nuclei would form more quickly, because in these conditions urea decomposes more rapidly, thus producing higher concentrations of NH_3_ and CO_3_^2−^, which are sources of Ce(OH)CO_3_ nuclei. Subsequently, nucleated Ce(OH)CO_3_ was transform to a CeO_2_ phase via calcination at high temperature. These phenomena occur in the order of the following equations (Cui et al., 2010):

$$H_{2}\mathrm{N-CO-N}H_{2}↔NH^{\mathrm{4+}}+\mathrm{OC}N^{-}$$ (8)

$$\mathrm{OC}N^{-}+OH^{-}+H_{2}O\to NH_{3}+CO_{3}^{\mathrm{2-}}$$ (9)

$${\left[\mathrm{Ce}\left(\mathrm{OH}\right){\left(H_{2}O\right)}_{\mathrm{n-1}}\right]}^{2+}+2CO_{3}^{\mathrm{2-}}\to \mathrm{CeOHC}O_{3}\left(\mathrm{CeC}O_{3}\mathrm{OH}\right)+\left(\mathrm{n-1}\right)H_{2}O$$ (10)

$$4\mathrm{Ce}\left(\mathrm{OH}\right)CO_{3}+O_{2}\to \mathrm{4CeC}O_{2}+4CO_{2}+2H_{2}O$$ (11)

The occurrence of the specified reactions could be confirmed by pH measurements conducted on the hydrothermally aged solutions. The pH values of the solutions were 7.6, 8.3, 10.6, and 10.4 for the hydrothermally aged solutions obtained at HT values of 120°C, 140°C, 160°C, and 180°C, respectively. This increase in the rate of urea decomposition associated with increases in the value of HT would explain the higher pH values measured after hydrothermal treatment in the case of CeO_2_160 with respect to CeO_2_140; as an additional result, a higher BET surface area for CeO_2_ would be observed for ceria prepared at higher HT values, even though the ceria would be composed of particles of larger size. Moreover, higher HT accelerates crystal formation with a lot of multiple grain boundaries. The aggregation of particles can cause collision and dislocation of grain boundaries and induce more defect formation. Thereby, the enhanced physical properties of CeO_2_180 contributed to greater chemical properties such as higher defect concentration and redox properties as confirmed by Raman and H_2_-TPR.

After Au deposition, differences in the structural properties of CeO_2_X samples resulted in significantly different characteristics for the Au/CeO_2_X catalysts. CeO_2_120 was characterized by a lower degree of particle agglomeration than the other three supports, as indicated by the relevant SEM and TEM images, as well as the lowest CeO_2_(111) fraction; therefore, Au/CeO_2_120 exhibited the smallest Au particle size, which accounted for Au/CeO_2_120 exhibiting the largest Au particle dispersion. This result was in line with previous reports that rod-shaped CeO_2_ particles are dominated by more active {110} and {100} surfaces that could provide the largest extent of Au dispersion for the various CeO_2_ morphologies (Huang et al., 2009; Yi et al., 2010; Eaimsumang et al., 2019). Each surface is characterized by a different energy, which defines the stability of the surface and the likelihood of formation of oxygen vacancies. Based on TEM evidence, small Au particles can effectively distribute on CeO_2_ (200) surface with respect to Au/CeO_2_120. In fact, larger Au particles tended to deposit on the more stable (111) surface. This evidence is indicative of the significant effect that the morphology and the exposed area of the CeO_2_ surface has on the extent of Au dispersion on the CeO_2_ support. In fact, the presence of Au NPs well-dispersed on CeO_2_120 resulted in improved catalytic properties, such as increased oxygen vacancy concentration and superior redox properties. The higher surface contact of small Au particles can effectively bind on a CeO_2_ surface, and consequently yield Au in the cationic state. The oxygen vacancies nearby an Au cluster can transfer an electron to the Au NP, which consequently is composed of electron rich Au NPs. Au^3+^ ions can replace Ce^4+^ ions in the ceria lattice to create an electron at the ceria surface. This could generate oxygen vacancies associated with the presence of Ce^3+^ centers close to Au sites. This results in a weakening of the positive potential around Au^3+^ ions, causing the Au 4f XP spectra to exhibit a negative shift (Shen et al., 2008). Furthermore, the weaker potential between the Au^3+^ also promoted reducibility of gold oxide at the very low reduction temperature of 89 °C, as evidenced by the characteristics of the H_2_-TPR profiles reported in Figure 5. Notably, the reduction temperature of the Au/CeO_2_ catalysts has been observed to also be related to the orientation of Au on the CeO_2_ support. Au NPs on a CeO_2_ support exhibited a reduction temperature lower than 100 °C. By contrast, multilayer Au exhibited two reduction peaks at 163 and 214 °C (Wang et al., 2015).

### 3.3 Catalytic performance

#### 3.3.1 CeO_2_X catalytic activity

**FIGURE 9:**
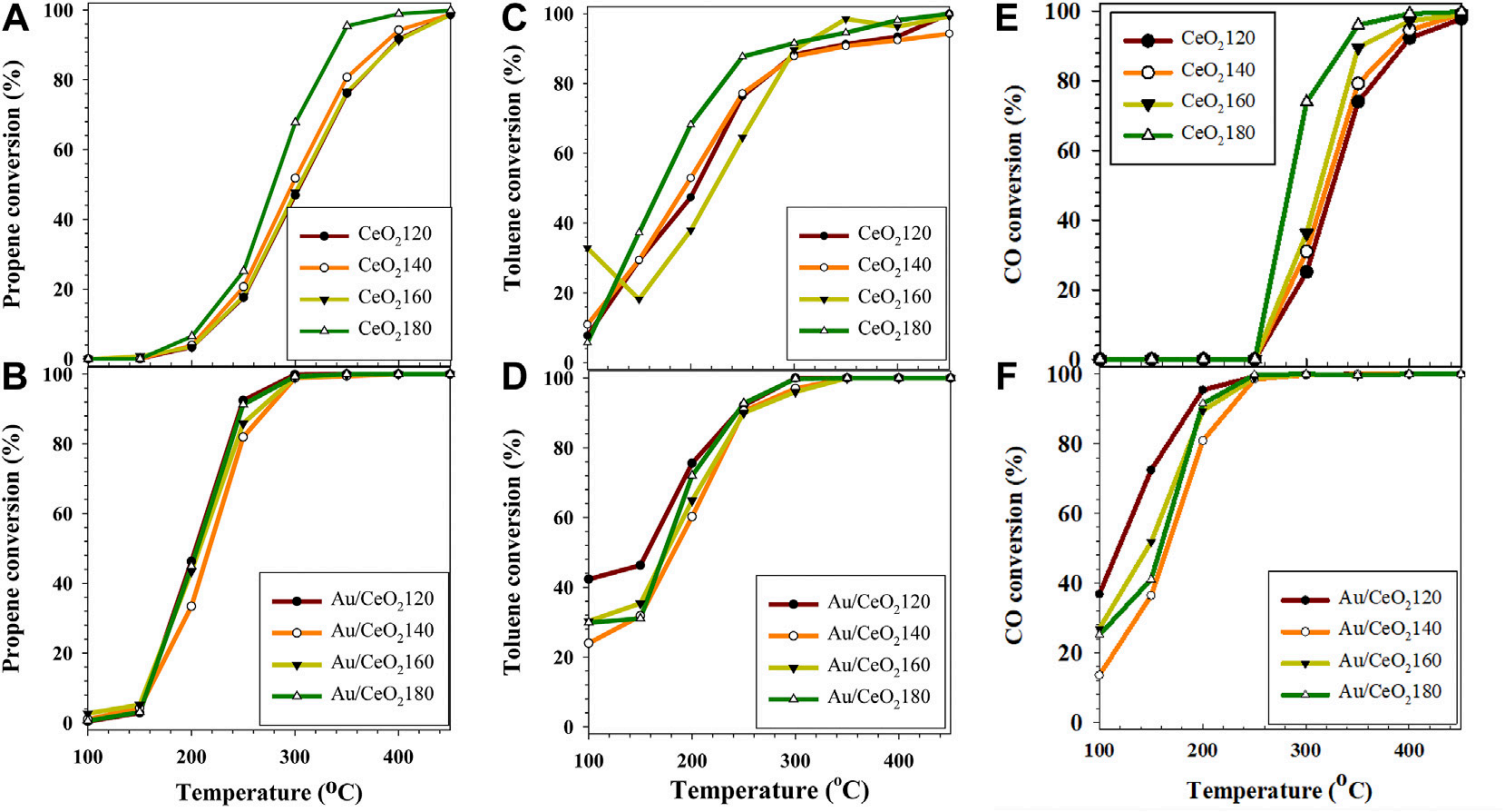
Catalytic activities of the CeO_2_X and Au/CeO_2_X catalysts for the oxidation of a propene/toluene/CO mixture at a gas hourly space velocity of 50,000 h^−1^ represented in terms of conversion level (%) of **(A,B)** propene **(C,D)** toluene, and **(E,F)** CO. CeO_2_X: cerium oxide obtained at a value X for the temperature of the preparatory hydrothermal process; Au/CeO_2_X: CeO_2_X-supported gold catalysts.

The CeO_2_X samples were tested for the simultaneous oxidation of the propene/toluene/CO gas mixture. The CeO_2_X samples were most active toward toluene oxidation. At the lowest reaction temperature (100 °C), 5.7–10.9% of toluene was removed over the CeO_2_X catalysts (Figure 9), values comparable to the 6.3% toluene removal measured over CeO_2_ supported on granular carbon (Trung et al., 2020). This observation in the present study was probably due to saturated toluene coverage of the CeO_2_ surface, which was indicative of toluene preferential adsorption onto the catalyst surface with respect to other reactants. The temperature when the conversion was 50% (T_50_) was also used as an indicator of catalytic activity. In order of increasing T_50_ of toluene conversion, the various CeO_2_X catalysts were (Table 4):

$$\mathrm{Ce}O_{2}\mathrm{180<Ce}O_{2}\mathrm{140<Ce}O_{2}\mathrm{120<Ce}O_{2}\mathrm{160}$$ (12)

**TABLE 4:** Reaction temperatures corresponding to 50% (T_50_) and 90% (T_90_) substrate conversion catalyzed by the CeO_2_X supports and Au/CeO_2_X catalysts in the oxidation of the propene/toluene/CO gas mixture.

Samples	T_50_ (°C)			T_90_ (°C)			Space Velocity	References
	Propene	Toluene	CO	Propene	Toluene	CO		
CeO_2_120	305	205	325	400	325	393	^a^50,000 h^−1^	This work
CeO_2_140	295	195	320	385	325	387	^a^50,000 h^−1^	This work
CeO_2_160	305	220	310	400	300	343	^a^50,000 h^−1^	This work
CeO_2_180	280	170	285	340	280	337	^a^50,000 h^−1^	This work
3%Au/CeO_2_120	210	160	119	248	243	188 (38^b^)	^a^50,000 h^−1^	This work
3%Au/CeO_2_140	215	180	165	274	249	227 (90^b^)	^a^50,000 h^−1^	This work
3%Au/CeO_2_160	210	170	146	266	250	202 (73^b^)	^a^50,000 h^−1^	This work
3%Au/CeO_2_180	210	170	146	248	243	198 (70^b^)	^a^50,000 h^−1^	This work
4%Au/CeO_2_	175	240	< 25	210	270	25	^b^60,000 ml/(g·h)	Aboukaïs et al. (2013)
1.3%Au/CeO_2_	152	208	N/A	175	270	N/A	^c^35,000 h^−1^	Ousmane et al. (2011)
4.1%Au/Al_2_O_3_	375	N/A	N/A	410	N/A	N/A	^d^9000 ml/(g·h)	Gluhoi et al. (2005)
1%Au/TiO_2_	332	367	N/A	340	>400	N/A	^e^60,000 ml/(g·h)	Hosseini et al. (2007)

*T_90_ of the independent CO, oxidation reaction.

a Reactant composition: 1,000 ppm propene, 100 ppm toluene, 1,000 ppm CO, 6% O_2_, N_2_ balance.

b Reactant composition: 6,000 ppm propene, 2000 ppm toluene, 1,000 ppm CO, air balance.

c Reactant composition:1,000 ppm propene, 1,000 ppm toluene 9% O_2_, he balance.

d Reactant composition: 1,200 ppm propene, 1.08% O_2,_ he balance.

e Reactant composition:1,000 ppm propene, 1,000 ppm toluene.

CeO_2_X began to catalyze propene oxidation at about 200 °C, and T_50_ is in agreement with the catalytic performance of CeO_2_ pretreated in O_2_/He at 500 °C prior to reaction (Delannoy et al., 2010). The rate of propene oxidation increased with the reaction temperature, and complete substrate conversion was obtained at 450 °C. In order of increasing T_50_ of propene oxidation, the various CeO_2_X catalysts can be ordered as follows:

$$\mathrm{Ce}O_{2}\mathrm{180<Ce}O_{2}\mathrm{140<Ce}O_{2}\mathrm{120}=\mathrm{Ce}O_{2}\mathrm{160}$$ (13)

Simultaneous CO oxidation showed high conversion, between 25.0 and 73.8% at 300 °C. CeO_2_180 exhibited better catalytic performance than the other CeO_2_X catalysts, which performed 90% conversion for CO oxidation at the low reaction temperature of 280 °C. The efficiency of toluene oxidation over CoAl(Ce) mixed oxides has been reported to be improved as a result of the presence of CO in the feed stream. It was claimed that heat released from CO oxidation can cause a local increase in the temperature of the catalyst bed, thus helping to accelerate toluene oxidation (Genty et al., 2019). The T_90_ of toluene was still lower than that of other reactants, in the 280–325°C range, with the lowest T_90_ for CeO_2_180.

Generally, CeO_2_ was observed to be active for CO oxidation alone at lower reaction temperatures (Zhang R. B. et al., 2017), than for mixed oxidation observed in our work. Propene has a stronger interaction with the catalyst surface than CO, since it is well known that propene forms a stronger Π-complex than CO as established on Au/TiO_2_ by DFT calculations (Chen et al., 2013). CO adsorbed on the catalyst surface might be replaced by propene. Hence, CO oxidation might be suppressed by propene.

The catalytic activity for VOC oxidation over CeO_2_ is likely correlated to the textural properties of CeO_2_; therefore, higher HT values could result in more advantageous physical properties of ceria, such as higher BET surface area. As a consequence, some chemical properties also improved, namely more readily reducible surface oxide, and higher defect concentration.

Although the surface reduction temperature of CeO_2_ was high, in the 551–561°C range, reducibility between surface to bulk ratio (H_2_ uptake between T_2_ and T_3_ in Table 2) was observed to improve in the following order:

$$\mathrm{Ce}O_{2}\mathrm{180}\left(0\mathrm{.55}\right)\mathrm{>Ce}O_{2}\mathrm{140}\left(0\mathrm{.48}\right)\mathrm{>Ce}O_{2}\mathrm{120}\left(0\mathrm{.46}\right)>\mathrm{Ce}O_{2}\mathrm{160}\left(0\mathrm{.44}\right)$$ (14)

The reduction process initially takes place at the CeO_2_ surface and then proceeds with reduction of the bulk. The increase in reducible surface area associated with increases in the HT values resulted in the broadening of the ceria reduction peak. The initial reduction temperature had a value of 303°C in the case of CeO_2_180. In contrast, the initial reduction temperature of CeO_2_120 was about 350°C. Moreover, the oxygen transport in the ceria lattice is important for formation of intrinsic defects. The defects can be generated by thermal disorder and interaction with the surrounding atmosphere (Agarwal et al., 2015). CeO_2_180 was characterized by the highest defect concentration, which agreed with the observation that CeO_2_180 exhibited a slightly higher lattice constant than the other CeO_2_X samples. The presence of such a large number of defects results in CeO_2_180 displaying a superior VOC oxidation activity with respect to the other CeO_2_X samples; notably, active oxygen released from the CeO_2_ structure was assumed to be the main oxidizing agent in the observed oxidation reaction and also implemented oxygen replacement at the defect sites to favor re-oxidation (Scirè et al., 2003; Liotta et al., 2008).

#### 3.3.2 Au/CeO_2_X catalytic activity

The addition of Auto CeO_2_ can enhance activity, due to availability of Au active sites and improved oxygen mobility by incorporation of Au. Evidence from several published studies indicate that VOC oxidation takes place over doped CeO_2_ catalysts *via* a Mars-van Krevelen (MvK) mechanism (Scirè et al., 2003; Andreeva et al., 2006; Delannoy et al., 2010; Aranda et al., 2012), whereby the reductant reacts with lattice oxygen of the metal oxide to form CO_2_, which is subsequently desorbed from the catalyst surface, resulting in the formation of oxygen vacancies. Subsequently, molecular oxygen (O_2_) is readily adsorbed on the vacant site and is eventually transformed to become the active oxygen species (O^2−^) in order to preserve charge balance (Liu et al., 2018). This emphasizes that the ability of the CeO_2_ support to be involved in oxygen exchange processes plays a crucial role in determining the catalyst activity for VOC oxidation.

Based on T_50_ for propene oxidation of the Au/CeO_2_X catalyst series, temperatures were lower (in the 70–95°C range) than those of the parent CeO_2_ catalysts. The light-off temperature was measured at 150°C, coincident with the value reported in the literature for 4 wt% Au/CeO_2_ prepared by the DP technique (Aboukaïs et al., 2013). The T_50_ value in the present study was higher than Pt and Pd catalysts. Notably, Pt and Pd catalysts can dissociatively adsorb oxygen on their surface to proceed *via* Langmuir–Hinshelwood mechanism more effectively than Au catalysts, so it is not surprising that they exhibit superior activity in catalyzing propene oxidation (Santos et al., 2010).

Au/CeO_2_X catalysts were very active for toluene total oxidation, and toluene was converted at the lowest temperatures. Overall, the T_50_ values for the Au/CeO_2_X catalysts decreased by 15–50°C with respect to the parent CeO_2_X supports. Au/CeO_2_120 was the most active catalyst, and it exhibited the lowest T_50_ value at 160°C. Moreover, toluene conversion catalyzed by Au/CeO_2_120 was significant between 100 and 200 °C. Initial toluene conversion was 42.3%, a value that was 1.4–1.8-fold higher than that of the other three Au/CeO_2_X catalysts. The catalytic activity of CO oxidation over the Au/CeO_2_X catalysts improved substantially with respect to CeO_X_, as all catalysts exhibited higher activity at 100°C (see Figure 7). In addition, the T_50_ value for CO oxidation of the Au/CeO_2_X catalysts was reduced by approximately 139–208°C with respect to bare ceria supports. Similar to observations for the catalytic conversion of toluene, AuCeO_2_120 exhibited significant catalytic activity for CO oxidation at temperatures between 100 and 200°C.

Many key factors contributed to determining the catalyst activities at low temperatures, including Au crystal size, defect concentration, Au oxidation state, and changes in the structural properties of CeO_2_ as a result of Au loading. The smallest Au crystallites were present on the surface of Au/CeO_2_120. CO can effectively bind to cationic Au (Au^n+^) and thus be in close proximity to react with neighboring active oxygen centers, leading to CO oxidation. Moreover, Au supported on structurally unstableCeO_2_ {100} planes preferentially bind more CO on the surface, activating CO, improving the reactivity for CO oxidation (Ha et al., 2018). The improved redox properties of the catalyst were also important. According to the MvK model, lattice oxygen participates in the oxidation reaction as the active oxygen species. The available defects act as active sites to activate oxygen, which is crucial to the progress of the reaction. Au/CeO_2_120 exhibited the highest concentration of surface oxygen vacancies among all Au/CeO_2_X catalysts, as determined from Ce^3+^ and O_defect_ surface concentrations measured through XPS analysis.

The oxygen vacancies in the bulk of Au/CeO_2_120 were also relatively high, as measured from Raman spectroscopy data. Only Au/CeO_2_120 was characterized by the presence of peroxide (O_2_^2−^) species, as indicated by the dominant peak at 832 cm^−1^ in the Raman spectrum. The existence of peroxide suggested the formation of oxygen vacancy defects and their decoration with released oxygen (Nottbohm and Hess, 2012). This species resulted from O_2_ binding to Au-Ce^3+^ sites, located on Au nanocluster supported on CeO_2_ (111) planes, which relate to a low-coordination Au site (Ha et al., 2018). The benefit of the adsorption of oxygen species onto oxygen vacancies was reported in a study focusing on the catalytic performance of CoO_3_–CeO_2_ for propene oxidation reactions taking place below 400 °C (Liotta et al., 2008). The adsorption process is correlated with the efficiency of propene oxidation, as indicated by the results of O_2_-temperature-programmed desorption investigations. Notably, Au/CeO_2_140 presented the lowest number of oxygen vacancies in both the surface and the bulk for all Au/CeO_2_X catalysts. This catalyst also exhibited the lowest CO conversion rate throughout the investigated temperature range. The ease of mobility of oxygen in CeO_2_120 was substantially improved as a result of Au incorporation. The H_2_-TPR profile of Au/CeO_2_ emphasizes the facile reducibility, which showed the lowest reduction temperature of 89 °C. The considerable shift of the reduction to such a low temperature could be ascribed to Au^+^ substitution into the ceria lattice, which would contain vacant sites of Ce^4+^ accompanied by the generation of oxygen vacancies and an increase in oxygen mobility (Zanella et al., 2004; Ousmane et al., 2011).

The nature of Au active species have been previously proposed to have a significant impact on the catalytic performance of Au catalysts; however, they depend on the reaction conditions and the optimum catalytic sites for each reaction (Carltonbird et al., 2018). The identity of the catalytically active species in supported Au catalysts is still a point of intense debate. Under CO oxidation/WGS reaction conditions, the dominant oxidation state of gold is Au^0^. On the other hand, Au^3+^ sites were observed during the WGS reaction and oxidic Au species (Au^1+^ or Au^3+^) were observed during CO oxidation. In oxidation reactions, a small amount of oxidized gold exhibited superior catalytic activity in the oxidation of HCHO (Shen et al., 2008); in isobutane oxidation over a temperature range of 100–500°C, low temperature activity is related to Au^+^ and high temperature activity is associated with Au^3+^. According to some reports, catalysts comprising Au^3+^ ions do not exhibit any catalytic activity in CO oxidation. XPS and oxidation activity evidence collected in the present study indicated that the oxidation activity increased with the fraction of cationic Au species.

When considering the T_90_, which presented 90% of reactants converted, the catalytic performance of Au/CeO_2_ catalysts substantially differed from their low temperature performance. Au/CeO_2_120 and Au/CeO_2_180 exhibited the lowest equivalent T_90_ of propene and toluene conversion among the Au/CeO_2_X catalysts. Nevertheless, Au/CeO_2_180 exhibited a higher T_90_ of CO conversion than Au/CeO_2_120, and the difference was 10°C. This observation indicated that the catalytic activity at high reaction temperature did not rely on only the nature of Au, but also the synergistic function between Au and CeO_2_ support. Although CeO_2_180 has the largest Au crystal size, it still exhibited relatively high reducibility and the highest defect concentration among the Au/CeO_2_X catalysts, as indicated by the H_2_ uptake for surface reduction and Raman spectroscopy results, respectively. Therefore, Au catalysts still exhibit high catalytic performance at high temperature. Overall, the catalytic performances of the Au/CeO_2_X systems were clearly more influenced by improvements in the properties of CeO_2_, influenced by introduction of Au, than by the ability of Au to directly catalyze the reaction. The highest catalytic performance was not displayed by the catalysts characterized by the highest Au content on the surface, but by the catalysts exhibiting the capability to activate oxygen. Nevertheless, the addition of Au can improve the mobility of the oxygen atoms in CeO_2_ as a result of the weakening of Ce–O bonds brought about by Au incorporation. In summary, it can be concluded that Au has a secondary role with respect to the support in Au/CeO_2_X reactivity at high temperature.

In a study similar to the present one, the catalytic oxidation efficiency of 4-wt% Au/CeO_2_-DP was tested against a gas mixture comprising propene (6,000 ppm), toluene (2000 ppm), and CO (1,000 ppm); results indicated excellent catalytic activity for propene and CO oxidation, due to the presence of highly dispersed Au nanoparticles only 3.9 nm in size (Aboukaïs et al., 2013). The T_50_ of propene conversion was approximately 170°C, and the T_90_ of CO oxidation was around 25°C. However, the T_50_ of toluene conversion at 250°C was higher than those determined in our work (160–180°C). This could be explained by different reactant concentrations and the Au particle size effect. The lower concentration of toluene in the gas mixture (100 ppm) and the larger Au particle size (8.6 nm) in the present work could result in easier adsorption of toluene on the catalyst surface, favoring the oxidation reaction.

In previously published studies, the catalytic performance of 1 wt% Au/CeO_2_ was investigated for propene and toluene oxidation, independently of each other (Ousmane et al., 2011). The T_50_ for propene conversion and the T_100_ for toluene conversion had values of 152 and 230°C, respectively. However, in the relevant experiments the GHSV was only 35,000 h^−1^, and the increase residence time will achieve higher conversion. The catalytic performance of Au/CeO_2_X catalysts prepared in the present work was compared with those of literature-reported catalysts, including 4.1 wt%Au/Al_2_O_3_ (Gluhoi et al., 2005) and 1%Au/TiO_2_ (Hosseini et al., 2007). 4.1 wt%Au/Al_2_O_3_ exhibited a very high T_50_ value of 375°C, even though its surface area was high (260 m^2^/g), which is one factor for enhancing catalytic oxidation. The Al_2_O_3_ support is not a reducible oxide support and demonstrated lower catalytic activity because of the inability of oxygen exchange to participate in the reaction. on the other hand, the T_50_ values of 1%Au/TiO_2_ were 332 and 367°C for the propene and toluene oxidation, respectively. These data imply that Au can facilitate oxygen mobility in the CeO_2_ structure by weakening Ce-O bond better than it can in TiO_2_, as a result of the strong interaction between Au and CeO_2_.

The catalytic performance of the Au/CeO_2_X catalyst series for the oxidation of CO alone was tested in order to investigate the effect of competing oxidation reactions. The Au/CeO_2_X catalysts were considerably more active toward CO oxidation in the presence of CO alone (T_90_: 38–90°C) than in the presence of the propene/toluene/CO gas mixture (T_90_: 188–227°C). However, the tentative relative order of the T_90_ values of CO oxidation in the presence of CO alone for the Au/CeO_2_X catalysts was the same as that determined in the case of the experiments conducted in the presence of the propene/toluene/CO gas mixture. Au/CeO_2_120 was characterized by the lowest T_90_ (38 °C; Table 4), while the order in increasing T_90_ for the other catalysts was:

$$\mathrm{Au}/\mathrm{Ce}O_{2}\mathrm{180}<\mathrm{Au}/\mathrm{Ce}O_{2}\mathrm{160}<\mathrm{Au}/\mathrm{Ce}O_{2}\mathrm{140}$$ (15)

These data indicate that, in the presence of propene and toluene, CO oxidation was readily hindered as a result of the competitive adsorption on the catalyst surface of reactants and of the relevant oxidation intermediates. Indeed, the inhibition of CO oxidation over a Pt/γ-Al_2_O_3_ catalyst in the presence of propene has also been reported (Hazlett and Epling, 2016). The partial oxidation of propene to ethene contributed to CO oxidation inhibition due to ethylene’s subsequent partial oxidation to acetate on the catalyst surface. Based on the overall catalytic results, Au supported on CeO_2_ characterized by bundled rod-like morphology (Au/CeO_2_120) exhibited the best catalytic activity for both reactions (*i.e.*, CO oxidation alone and CO oxidation in the presence of propene and toluene). Although Au/CeO_2_140 also possessed a bundle rod-like morphology, the diameter of each rod was larger for this catalyst than for Au/CeO_2_120, having a lower rod aspect ratio. Consequently, Au dispersion is lower for Au/CeO_2_140, resulting in lower catalytic performance. Moreover, Au/CeO_2_140 still contains more pores in the micropore region. Hence, the diffusion of all reactants (propene, toluene, and CO) at the same time into the pore channel may be hindered.

## 4 Conclusion

The temperature (HT) at which CeO_2_ is synthesized from cerium hydroxycarbonate by a hydrothermal method significantly influences ceria morphology and physicochemical properties. Particle size increased with increasing HT, and so did, as a consequence, the crystal phase ratio (I_111_/I_200_), as determined by XRD. Therefore, CeO_2_120, which was determined to be composed of bundles of rod-like morphology, exhibited the lowest value for the I_111_/I_200_ ratio, as well as the most favorable properties for catalysis, such as the highest Au particle dispersion, the most advantageous redox properties at low temperature, and the highest number of oxygen vacancies formed on the catalyst surface and in the bulk. The catalytic activity for the oxidation of a propene/toluene/CO mixture at low temperature was positively influenced by an increase in the number of oxygen vacancies and an increase in the concentration of Ce^3+^ ions on the catalyst surface, as well as by a decrease in reduction temperature. Toluene oxidation was the reaction that occurred most easily in the mixture, evident by the lowest T_50_ value. The T_90_ of CO conversion in the existence of propene/toluene/CO mixture was significantly higher than that obtained for independent CO oxidation. Au/CeO_2_120 was the most active catalyst for the oxidation of the propene/toluene/CO gas mixture as well as the oxidation of CO alone, which also related to the observation that the smaller Au nanoparticles can provide more reactive sites at the perimeter and largely enhanced oxygen mobility and oxygen vacancies, including the presence of highly cationic gold species.

## Data Availability

The raw data supporting the conclusion of this article will be made available by the authors, without undue reservation.

## Abbreviations

BE, Binding energy; GHSV, Gas hourly space velocity; HT, Hydrothermal temperatures; LPG, Liquefied Petroleum gas; MvK, Mars van Krevelen; TCD, Thermal conductivity detector; VOC, Volatile organic compounds; XPS, X-ray photoelectron spectroscopy; XRD, X-ray diffraction.
