# Supplementary material for: Relationship between hydrothermal temperatures and structural properties of CeO2 and enhanced catalytic activity of propene/toluene/CO oxidation by Au/CeO2 catalysts
Source: Front Chem. 2022 Sep 21;10:959152. doi: 10.3389/fchem.2022.959152 (PMC9532521; doi:10.3389/fchem.2022.959152)
Supplement: Supplementary file 1 [file DataSheet1.docx]

**Supplementary Information**

**Fig. S1** BJH pore size distribution of the CeO_2_X supports and Au/CeO_2_X catalysts. CeO_2_X: cerium oxide obtained at a value X for the temperature of the preparatory hydrothermal process; Au/CeO_2_X: CeO_2_X-supported gold catalysts.

**Fig. S2** Representative Ce 3d X-ray photoelectron spectra of Au/CeO_2_X catalysts. CeO_2_X: cerium oxide obtained at a value X for the temperature of the preparatory hydrothermal process; Au/CeO_2_X: CeO_2_X-supported gold catalysts.
